# Predictors of in-hospital mortality in older patients undergoing distal femur fracture surgery: A case-control study

**DOI:** 10.1051/sicotj/2023035

**Published:** 2023-12-07

**Authors:** Ting-An Cheng, Po-Hsuan Lai, Hao-Chun Chuang, Kai-Lan Hsu, Fa-Chuan Kuan, Wei-Ren Su, Chih-Kai Hong

**Affiliations:** 1 Department of Orthopaedic Surgery, National Cheng Kung University Hospital, College of Medicine, National Cheng Kung University Tainan Taiwan; 2 Department of Family Medicine and Community Medicine, E-Da Hospital, I-Shou University Kaohsiung Taiwan; 3 Musculoskeletal Research Center, Innovation Headquarter, National Cheng Kung University Tainan Taiwan; 4 Department of Orthopaedic Surgery, National Cheng Kung University Hospital, Dou-Liou Branch, College of Medicine, National Cheng Kung University Yunlin Taiwan

**Keywords:** In hospital mortality, Geriatric fracture, Distal femur fracture, Risk factor, Delay to surgery

## Abstract

*Introduction*: Geriatric fractures including distal femur and hip fractures are associated with high mortality rates. Currently, prognostic factors for in-hospital postoperative mortality are not identified. We aimed to evaluate overall in-hospital mortality and related potential risk factors in elderly patients who underwent distal femur fracture surgery. *Materials and methods*: A retrospective cohort study of patients older than 60 years, who underwent distal femur fracture surgery between January 01, 2003, and December 31, 2021, was conducted. A case-control study was conducted to compare two age-matched groups of elderly patients of equivalent ages at a 1:4 ratio. The in-hospital mortality rate was calculated and potential confounders were compared between groups. *Results*: A total of 170 patients were enrolled; five died during hospital stay after undergoing surgery, yielding a 2.94% in-hospital mortality rate. Twenty patients who did not die were included in the control group. Patients’ demographics were similar. The case-control comparison showed that the time from injury to surgery, preoperative hemoglobin level, estimated glomerular filtration rate (eGFR), and white blood cell count were significant factors correlated with in-hospital mortality. *Discussion*: The overall in-hospital mortality rate was 2.94%. Significant risk factors for in-hospital mortality included a longer time from injury to surgery, lower preoperative hemoglobin level and eGFR, and higher preoperative white blood cell count. In conclusion, preoperative comprehensive geriatric assessment, including cognitive, nutritional, and frailty status, should also be considered in the elderly fracture care model.

## Introduction

Distal femur fractures account for 1% of all fractures and about 4–6% of femoral fractures [1, 2]. The number of distal femur fractures increases with age, with an incidence of 4–9%/100,000/year. In the elderly population, distal femur fractures are the second most common fracture of the femur after hip fractures [3, 4].

Geriatric fractures, such as hip and distal femur fractures, are known as osteoporotic and fragile fractures in the elderly [3, 5–8]. Geriatric fractures are associated with a high mortality rate [1, 7–13]. The in-hospital mortality following hip fracture surgery is approximately 2% [11, 12]. Previous studies have also indicated that the overall mortality of distal femur fractures is even higher than that of hip fractures [1, 7, 9, 10]. Tsai et al. reported an 8.3% overall in-hospital mortality in elderly patients after distal femur fracture surgery [1].

Although the overall mortality rate of geriatric distal femur fractures had been well established, the prognostic factors associated with the in-hospital mortality rate of distal femur fractures after surgery have not been identified [1]. Hence, the primary aim of this study is to evaluate the overall in-hospital mortality in elderly patients after distal femur fracture surgery. The secondary aim is to identify the potential risk factors of in-hospital mortality in elderly patients after distal femur fracture surgery.

## Material and methods

### Patient enrollment

This study was approved by the Institutional Review Board of the National Medical Center (IRB No. B-ER-112-020). This retrospective study was conducted through a medical chart review from January 2013 to December 2021 at National Cheng Kung University Hospital, Tainan, Taiwan. The inclusion criteria were as follows: (1) patients older than 60 years, (2) patients diagnosed with distal femur fractures, and (3) patients who underwent surgical fixation, including open reduction and internal fixation or external skeletal fixation. Patients who underwent surgery for (1) periprosthetic fracture, (2) infection, (3) osteoarthritis, or (4) tumor metastasis were excluded. Finally, 170 geriatric patients over 60 years old with a diagnosis of distal femur fractures were enrolled. All patients were followed until their discharge. Among the 170 patients with distal femur fractures, five died during the hospital stay after undergoing surgery. Of the in-hospital mortality group, the mean age was 84.6 ± 12.4 years (range 67–98 years) and the average postoperative survival period was 19.4 ± 22.9 days (range 3–59 days). The causes of death in these five patients included gastrointestinal bleeding, pneumonia, acute myocardial infarction, and sepsis (*n* = 2). After cross-matching by age with a 1:4 ratio, 20 patients who underwent distal femur fracture surgery and survived were considered in the control group; these patients had a mean age was 82 ± 9.11 years.

### Measurement of parameters

We collected demographic data, including age; sex; body mass index (BMI); American Society of Anesthesiologists (ASA) classification; underlying comorbidities; and laboratory data, such as preoperative and postoperative hemoglobin, sodium, and creatinine levels; estimated glomerular filtration rate (eGFR); platelet count; and white blood cell count. The time from injury to operation, fracture pattern, intraoperative blood loss, type of anesthesia, and duration of operation were also recorded.

### Statistics

Patient characteristics and mortality data were extracted from electronic medical charts.

This case-control study was conducted to compare two age-matched groups of elderly patients of equivalent ages but different survival outcomes after distal femur fracture surgery [11]. We cross-matched by age in a 1:4 ratio. Five patients died during the hospitalization, and 20 patients who survived were included in the control group.

Statistical analysis was performed to determine the differences between the two groups using the Mann–Whitney *U* test for parametric data and the chi-square test (or Fisher’s exact test, as appropriate) for non-parametric data. Quantitative variables are shown as means and standard deviations and qualitative variables as frequencies and percentages. After the primary analyses, factors with an alpha value of <0.1 were included in a conditional logistic regression analysis using a generalized linear model with generalized equivalent equations (GEE), which examined the relative contribution of each potential predictor to the incidence of death. Each case and its corresponding four controls comprised a stratum, and the factors were analyzed as within-subject variables. The adjusted odds ratios (AORs) and 95% confidence intervals (95% CIs) of the variables were calculated, and the significance of the AOR was tested using the Wald Chi-square test. A two-tailed *P*-value of 0.05 was considered statistically significant in the conditional logistic regression analysis. All data were analyzed using SPSS version 17 (IBM, Armonk, NY, USA).

## Results

The overall in-hospital mortality rate was 2.94%. The demographic data of these two cohorts were listed in Table 1, and no significant difference was observed in terms of sex, age, BMI, ASA classification, platelet count, postoperative hemoglobin level, sodium level, fracture pattern, fracture location, type of anesthesia, intraoperative blood loss, or duration of the operation.

**Table 1:** Comparison of clinical data by study groups.

	In-hospital mortality (*n* = 5)	Control (*n* = 20)	*P* value
Age	84.6 ± 12.4	82 ± 9.1	.371
Gender			
Male	2 (40%)	3 (15%)	
Female	3 (60%)	17 (85%)	
BMI (kg/m^2^)	20.8 ± 2.2	24 ± 5.2	.129
Fracture pattern			.162
A	3 (60%)	14 (70%)	
B	2 (40%)	2 (10%)	
C	0	4 (20%)	
Location			
Right	3 (60%)	8 (40%)	
Left	2 (40%)	12 (60%)	
Time from injury to surgery (h)	199.4 ± 162.57	24.7 ± 22.3	**<.001**
Type of surgery			.145
Plating	3 (60%)	17 (85%)	
K-wires	1 (10%)	0	
TKA and screws	1 (10%)	0	
TENS	0	1 (5%)	
ESF and wires	0	1 (5%)	
Screws	0	1 (5%)	
Blood loss (ml)	262.5 ± 149.3 (*n* = 4)	308.33 ± 193.4 (*n* = 18)	.837
Surgical time (min)	126.8 ± 67.9	184.4 ± 105.6	.243
ASA classification			.067
1	0	0	
2	0	5 (25%)	
3	3 (60%)	13 (65%)	
4	2 (40%)	2 (10%)	
Anesthesia type			.173
Spinal	1 (20%)	9 (45%)	
Endotracheal tube intubation	2 (40%)	9 (45%)	
Laryngeal mask	2 (40%)	1 (5%)	
Local	0	1 (5%)	
Underlying comorbidities			
Hypertension	4 (80%)	17 (85%)	.785
Diabetes mellitus	2 (40%)	5 (25%)	.504
Coronary artery disease	1 (20%)	2 (10%)	.538
Chronic kidney disease	2 (40%)	5 (25%)	**.018**
III a	0	3	
III b	0	2	
IV	0	0	
V	2	0	
Cerebral vascular accident	0	0	n/a
Peptic ulcer history	1 (20%)	1 (5%)	.367
Viral hepatitis	0	0	n/a
Dementia	1 (20%)	3 (15%)	.785
Affective or psychotic disorder	0	0	n/a
Malignant cancer history	0	4 (20%)	.549
Charlson comorbidity index	5.2 ± 1.0	4.4 ± 1.4	.192
Cardiac echo (%)	70.5 ± 4.6 (*n* = 3)	70.4 ± 8.9 (*n* = 17)	.976
Laboratory data			
Na (mmol/L)	138.4 ± 5.8	138.4 ± 4.2	.818
Preoperative Hb	6.7 ± 1.7	10.9 ± 2.2	**.001**
Postoperative Hb	8.6 ± 2.1	9.1 ± 1.5	.869
Platelet count	183.0 ± 103.2	192.5 ± 42.4	.767
White blood cell count	11.4 ± 2.5	8.4 ± 2.3	**.019**
Serum creatinine (mg/dL)	3.7 ± 4.6	0.7 ± 0.2	.060
eGFR	41.8 ± 32.2	78.7 ± 22.1	**.024**

Variables are presented as mean and standard deviation or frequencies and percentages.

*P*-value, based on the Mann–Whitney *U* test for parametric data and the Chi-square test or the Fisher’s exact test when appropriate for non-parametric data.

A *P* < 0.05 was displayed in bold fonts.

Abbreviations: ASA – American Society of Anesthesiology; eGFR – estimated glomerular filtration rate; Hb – hemoglobin.

Preoperative hemoglobin level, eGFR, and white blood cell count were significantly related to the in-hospital mortality rate. The Mann–Whitney *U* test revealed that preoperative hemoglobin level (6.72 ± 1.8 vs. 10.94 ± 2.2 g/dL, *p* = 0.001), white blood cell counts (11,460 ± 2510 vs. 84,000 ± 2350/μL, *p* = 0.019), eGFR (41.88 ± 32.23 vs. 78.75 ± 22.14, *p* = 0.024), and time from injury to operation (199.4 ± 162.57 vs. 24.7 ± 22.3 min, *p* < 0.001) were significantly different between these two groups. Therefore, these factors were included in a generalized linear model with a GEE for further regression analyses. Moreover, more patients with end-stage renal disease were identified in the mortality group than those in the control group. According to the GEE, the in-hospital mortality rate significantly increased by 4.6% for every hour delay from injury to surgery (ORs = 1.046, *p* = 0.005). Nevertheless, the in-hospital mortality rate significantly decreased by 5.3% for every increase in eGFR of 1 mL/min/1.73 m^2^ (ORs = 0.947, *p* = 0.032).

## Discussion

Previous studies indicated that geriatric fractures were related to a high mortality rate [1, 7–13]. Although a high mortality rate of distal femur fractures has been reported previously, the identification of factors associated with in-hospital mortality is lacking [1, 9, 10]. The current study identified several predictors related to in-hospital mortality after distal femur fracture surgery. This study majorly showed that the overall in-hospital mortality rate was 2.94% and the time from injury to surgery, preoperative hemoglobin level, eGFR, and white blood cell count were related to the in-hospital mortality rate. These factors could potentially be used to identify high-risk patients in the future.

The present study has several limitations. First, it was a retrospective study. Second, only five in-hospital mortality cases were collected, and the results do not represent the overall condition of patients with distal femur fractures. Third, this study included only patients who underwent distal femur fracture surgery. Patients who underwent conservative treatment or were not suitable for surgery were excluded, which may have caused selective bias and influenced the overall mortality rate. Finally, data were extracted from electronic medical records, which restricted us from collecting other important clinical parameters, such as pre-injury ambulatory status, mental conditions, residence type, and the use of medication. Further investigations with larger sample sizes are warranted.

The high mortality rates after distal femur fractures have attracted clinical attention [1, 9, 10]. It has been reported that the overall one-year mortality rate of distal femur fractures ranged from 10% to 38% [7, 9, 10, 14, 15]. Although several studies focused on the one-year mortality rate, only a few studies reported in-hospital mortality in patients with distal femur fractures [1]. Tsai et al. collected 26,325 patients over 65 years with distal femur fractures and found that 2206 died during hospitalization, resulting in an overall in-hospital mortality rate of 8.3% [1]. In the present study, the overall in-hospital mortality rate was 2.94%, which was slightly lower than that in the previous study [1]. The possible reason for the aforementioned difference was that patients admitted for conservative treatments for distal femur fracture were not enrolled in the present study. Differences in the inclusion criteria led to different mortality rates.

Delayed time from injury to surgery was significantly associated with an increased mortality rate in geriatric distal femur fractures (Table 2) [1, 10, 14, 15]. The overall mortality rate increased for patients who had a surgical delay of >2 days compared with patients who underwent surgery within 48 h after admission [10, 14, 15]. Similar to findings of previous studies, [1, 10, 14, 15] the time from injury to operation in the in-hospital mortality group was significantly greater than that in the control group in the present study. The present study further performed a general estimation equation and indicated that the in-hospital mortality rate increased to 4.6% for every delayed hour from injury to operation. Therefore, we suggested that geriatric patients with distal femur fractures should be treated surgically as soon as possible after the completion of the comprehensive preoperative survey.

**Table 2:** Common risk factors related to mortality after fractures in the literature.

Study	Patient number	Patients	Risk factors	Summary
Myers et al. [10]	*N* = 283	Geriatric distal femur (>60 y/o)	Surgical delay	A surgical treatment >2 days after injury was associated with increased patient mortality.
Moloney et al. [15]	*N* = 176	Geriatric distal femur (>60 y/o)	Surgical delay	One-year mortality was significantly associated with a delay in time to surgery >2 days.
Streubel et al. [14]	*N* = 44	Geriatric distal femur (>60 y/o)	Surgical delay	Surgical delay >4 days increased the 6-month and 1-year mortality risks.
Sheikh et al. [12]	*N* = 1356	Geriatric hip	Hb	Admission hemoglobin of <10 g/dL predisposed to 30-day mortality after hip fracture surgery.
Yombi et al. [16]	*N* = 829	Geriatric hip	Hb	Low hemoglobin at admission (<12 g/dL) was significantly associated with short- and long-term mortality after fragility hip fracture surgery.
Manosroi et al. [17]	*N* = 226	Geriatric hip (>50 y/o)	Hb	Increase of preoperative hemoglobin levels to ≥10 g/dL had an association with a 50% reduction in 1-year mortality among osteoporotic hip fracture patients.
Chiang et al. [11]	*N* = 817	Geriatric hip (>60 y/o)	Egfr	Poor renal function was a significant age-independent risk factor for in-hospital mortality in older adults undergoing hip fracture surgery.
Mandai et al. [21]	*N* = 9320	Hip, spine, forearm, upper arm, and leg (distal femur and proximal tibia)	ESRD	Hemodialysis patients experienced a 4.8-fold higher mortality rate after fractures than the general population.
Heffernan et al. [19]	*N* = 2448	Trauma (ISS ≥ 15)	WBC	Persistently abnormal CBC responses were associated with a higher mortality following trauma.
Santucci et al. [20]	*N* = 279	Blunt trauma	WBC	A significant elevation in WBC in a blunt trauma patient should heighten suspicion of occult injury.

Hb – hemoglobin; eGFR – estimated glomerular filtration rate; ESRD – end-stage renal disease; ISS – injury severity score; WBC – white blood cells; CBC – complete blood count.

An important finding of the present study was that the preoperative hemoglobin level, eGFR, and white blood cell count were significant risk factors for in-hospital mortality. Previous studies have reported that a low preoperative hemoglobin level is associated with mortality in geriatric hip fractures (Table 2) [12, 16–18]. The present study further indicated that this phenomenon could also be observed in geriatric distal femur fractures. Additionally, preoperative white blood cell count was also an important factor in predicting mortality [19, 20]. Previous studies indicated that a high white blood cell count or persistent leukocytosis after trauma was related to a greater mortality risk (Table 2) [19, 20]. This phenomenon was also observed in the geriatric distal femur fracture cohort, and the white blood cell count in the mortality group was significantly greater than that in the control group. Chronic kidney disease (CKD), which is a serious disease, also seems to be related to the mortality rate in geriatric patients with fractures (Table 2) [11, 21]. A previous study revealed that hemodialysis patients experienced a 4.8-fold higher mortality rate after fractures than that of the general population [21]. Similar findings were found in geriatric distal femur fractures in the present study; three out of five (60%) patients who died had CKD stage ≥3 and two of those patients were undergoing regular hemodialysis treatment. We further found that the in-hospital mortality rate significantly decreased by 5.3% for every 1 mL/min/1.73 m^2^ increase in eGFR. In summary, as several preoperative laboratory findings, including lower hemoglobin level, low eGFR, and higher white blood cell count, were related to in-hospital mortality, physicians should pay particular attention to high-risk patients who undergo surgical treatment for geriatric distal femur fractures.

Further, CKD was previously associated with geriatric syndromes, such as frailty, impaired nutritional status, and comorbidities, worsening their clinical outcomes [22, 23]. Anemia was also a predictive factor of mortality in the elderly and also possible presentation of nutritional deficiency or an indicator of cognitive decline [24]. Symptoms of anemia, such as fatigue, dyspnea, chest pain, and dizziness, also contribute to disability or functional outcomes of rehabilitation [25]. In geriatric fractures, frailty, malnutrition, and sarcopenia are proven to impart negative effects on clinical outcomes after fracture or surgery [26, 27]. Therefore, comprehensive geriatric assessment before distal femur surgery is important; this practice has been proven to shorten hospital stay days, unnecessary consultation time and wait, and preparation time for operating conditions.

In conclusion, the overall in-hospital mortality rate of patients who underwent distal femur fracture surgery was 2.94%. Significant risk factors for in-hospital mortality included a longer time from injury to surgery, lower preoperative hemoglobin level and eGFR, and higher preoperative white blood cell count. Preoperative comprehensive geriatric assessment, including cognitive, nutritional, and frailty status, should also be considered while implementing the elderly fracture care model.
